# Reply: Elevated serum matrix metalloproteinase 9 (MMP-9) concentration predicts the presence of colorectal neoplasia in symptomatic patients

**DOI:** 10.1038/sj.bjc.6604339

**Published:** 2008-04-15

**Authors:** N G Hurst

**Affiliations:** 1 Department of Surgery, Derby City General Hospital, Uttoxeter Road, Derby DE22 3NE, UK; 2 Institute for Cancer Studies, University of Birmingham, Birmingham B15 2TT, UK

**Sir**,

We thank the correspondents for their interest in our paper and for their constructive comments.

With regard to generation of the model for prediction of neoplasia, our model was based on all patients in the study, not restricted to disease-free patients as suggested by Belobrajdic *et al*, using raw sMMP9 concentrations rather than age-adjusted values. Age was a significant factor in risk of neoplasia and hence, the final risk was adjusted through the regression model applied.

Prospective prediction of the presence of neoplasia was attempted, as this is a more clinically relevant exercise in the pursuit of a non-invasive screening test for colorectal neoplasia. Improved accuracy through regression analysis was performed to direct future studies aimed at more accurate prediction in populations more closely approximating screening-target groups, rather than the high-prevalence group investigated in this preliminary study. We agree the *R*^2^ value of 0.027 is remarkably small and, if accurate, would have contradicted the assertion of a relationship between sMMP9 concentration and patient age. Unfortunately, the value reported is a misprint (as Belobrajdic *et al*, correctly state), which all authors failed to identify during proof reading and instead should have read *R*^2^=0.44. We apologise for the error.

We agree that maximum availability of data to the wider audience is desirable and did indeed exclude the suggested age versus sMMP9 concentration through consideration of space constraints. Again, box-plots of sMMP9 concentrations for individual subgroups were similarly excluded from the final draft for reasons of conciseness and brevity. We would welcome contact from groups with similar and comparable data sets in this field for purposes of combined analysis.

