# Reply: Elevated serum matrix metalloproteinase 9 (MMP-9) concentration predicts the presence of colorectal neoplasia in symptomatic patients

**DOI:** 10.1038/sj.bjc.6604543

**Published:** 2008-07-29

**Authors:** N G Hurst, D D Stocken, S Wilson, C Keh, M J O Wakelam, T Ismail

**Correction to**: *British Journal of Cancer* (2008) **98**, 1594. doi: 10.1038/sj.bjc.6604339

The authors of the above Reply to Letter to the Editor, originally published in volume no. **98**, issue 9, have requested a corrigendum to show the complete author list for the letter. This is now shown above.

